# Thermal conductivity measurements of proton-heated warm dense aluminum

**DOI:** 10.1038/s41598-017-07173-0

**Published:** 2017-08-01

**Authors:** A. McKelvey, G. E. Kemp, P. A. Sterne, A. Fernandez-Panella, R. Shepherd, M. Marinak, A. Link, G. W. Collins, H. Sio, J. King, R. R. Freeman, R. Hua, C. McGuffey, J. Kim, F. N. Beg, Y. Ping

**Affiliations:** 1 Lawrence Livermore National Laboratory, Livermore, CA 94550 USA; 2 University of Michigan, Nuclear Engineering Department, Ann Arbor, MI 48109 USA; 3 Massachusetts Institute of Technology, Plasma Science and Fusion Center, Cambridge, MA 02139 USA; 4 The Ohio State University, Physics Department, Columbus, Ohio 43210 USA; 5 University of California San Diego, Center for Energy Research, La Jolla, CA 92093 USA

## Abstract

Thermal conductivity is one of the most crucial physical properties of matter when it comes to understanding heat transport, hydrodynamic evolution, and energy balance in systems ranging from astrophysical objects to fusion plasmas. In the warm dense matter regime, experimental data are very scarce so that many theoretical models remain untested. Here we present the first thermal conductivity measurements of aluminum at 0.5–2.7 g/cc and 2–10 eV, using a recently developed platform of differential heating. A temperature gradient is induced in a Au/Al dual-layer target by proton heating, and subsequent heat flow from the hotter Au to the Al rear surface is detected by two simultaneous time-resolved diagnostics. A systematic data set allows for constraining both thermal conductivity and equation-of-state models. Simulations using Purgatorio model or Sesame S27314 for Al thermal conductivity and LEOS for Au/Al release equation-of-state show good agreement with data after 15 ps. Discrepancy still exists at early time 0–15 ps, likely due to non-equilibrium conditions.

## Introduction

Warm dense matter (WDM) is a regime where the Coulomb coupling parameter and the electron degeneracy parameter are of order one and matter cannot be well described with either condensed matter or ideal plasma theories. This loosely defined temperature-density phase space of approximately 0.1 to 100 electron volts and 0.1 to 20 g/cc is applicable to many astrophysical, geophysical, and laboratory plasmas, and therefore is of broad interest in multiple scientific communities^1^. Many systems either exist in this phase space or pass through it during their evolutions, and accurate models are vital to developing predictive capability of material properties. One of such fundamental properties is thermal conductivity, a transport coefficient that is a necessary input in every hydrodynamic code. There are a wide range of models on thermal conductivity, from the classical Spitzer formula^2^, commonly used Lee-More model^3–5^, to recently developed average-atom models^6–9^, molecular dynamics simulations^10, 11^ and density function theory calculations^12–14^. Predictions of thermal conductivities using different models vary substantially in the WDM regime^13, 15–17^. Experimental measurements are challenging yet especially needed to benchmark and validate these models.

Aluminum is a prototype material that is commonly used in model development. The early work on electrical conductivity measurements of Al through self-reflectivity^18^ is followed by non-equilibrium models suggesting rescaling of the temperature^19^, different data interpretation taking into account gradients^20^, and calculations showing resistivity saturation^21^. Recent advancement in both facilities and diagnostics have enabled more measurements on warm dense aluminum, including ionization potential depression^22, 23^, collision rates^24, 25^, and complex collision frequency^26^. Electrical conductivity has been measured at 0.02–0.5 g/cc^27^ or obtained from the complex collision frequency at solid density using a generalized Drude expression^26^. Although it is generally true that electrical conductivity is linked to thermal conductivity via the Wiedemann-Franz law, in the WDM regime the Lorentz number becomes temperature-dependent^28^ as a transition from the degenerate limit ${\pi }^{2}\mathrm{/3}$ to the Spitzer limit 1.622^29^. Direct measurements of thermal conductivity are still lacking for Al in the WDM regime.

In this paper we present the first experimental data on thermal conductivity of aluminum in a density range of 0.5–2.7 g/cc and a temperature range of 2–10 eV. The measurements employ a recently proposed platform of differential heating by laser-generated MeV protons^30^. A temperature gradient is initiated between Au and Al due to different energy deposition in these two materials^31^. The subsequent heat flow from hotter Au to Al rear surface is detected by two time-resolved optical diagnostics with ps time resolution. Using layered targets of Au only and Au with varying Al thickness, we are able to test both the release equation-of-state (EOS) and thermal conductivity models for Au and Al by comparing time-resolved data with hydrodynamic simulations.

## Results

### Experiments

**Figure 1:**
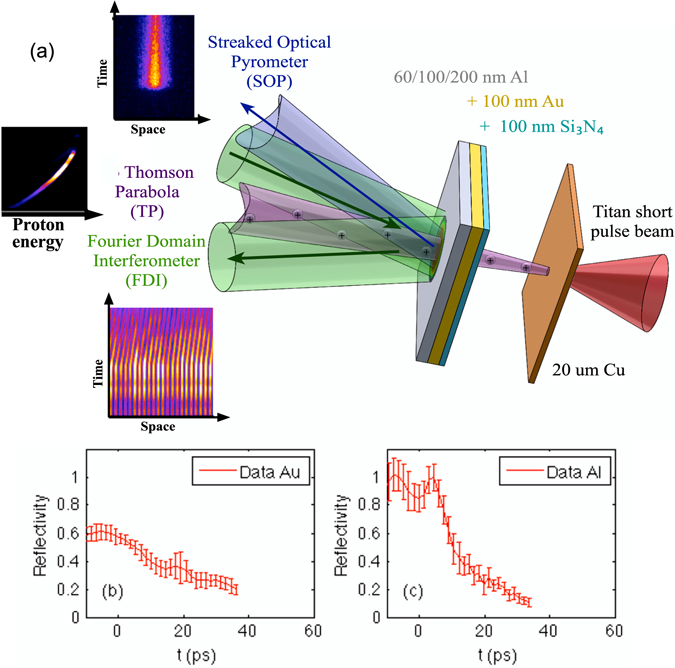
(**a**) Experimental setup for proton heating of multi-layer targets. Representative data of the SOP and FDI can be seen in the top and bottom left. The FDI beams were incident at 16 degrees from target normal in the horizontal plane and the SOP was measured at 16 degrees from target normal in the vertical plane. The plots are the measured reflectivity at 527 nm vs time for Au only (**b**) and Al targets (**c**).

The experiment was performed on the Titan laser at the Jupiter laser facility (JLF) at Lawrence Livermore National Laboratory (LLNL). The setup is shown in Fig. 1. A 0.7 ps laser pulse at 1053 nm was focused into a ~10 *μ*m spot with energies ranging from 15 to 30 joules for intensities on the order of 10^19^ W/cm^2^ on a 20 *μ*m Cu foil to generate proton beams via the target-normal-sheath acceleration (TNSA)^32^. A probe beam containing ~10 mJ of energy was frequency doubled to 527 nm and chirped to 50 ps for single-shot Fourier domain interferometry (FDI)^33^, providing time history of both reflectivity and phase shift. The proton energy spectrum was recorded every shot by a Thomson parabola (TP) ion spectrometer.

This work represents an implementation of a novel method^30^ of using protons to differentially heat a multi-layer target and study transport properties in strongly coupled plasmas. Proton heating has been demonstrated for efficient volumetric heating^34, 35^. Here we utilize the dissimilar atomic numbers of two materials to establish a temperature gradient for thermal conduction study. The main target consisting of Au and Al layers is located 50 *μ*m away from the proton-generating Cu foil. Both the 100 nm Au and 60, 100, and 200 nm of Al were deposited on a 100 nm Si_3_N_4_ substrate to ensure an optical-quality surface for FDI measurements. The Al thickness is chosen to be optically thick for pyrometric measurements yet still thin enough so that the thermal energy from the Au layer can traverse the Al layer and be detected before hydrodynamic cooling becomes dominant.

The thermal conduction heat flow is diagnosed as a time-resolved thermal emission at the heated Al rear surface by a streaked optical pyrometer (SOP) at 400 nm with 70 nm bandwidth. The time resolved reflectivity from FDI was used to determine the emissivity for grey-body temperature correction through the Kirchhoff’s law under the assumption of local thermal equilibrium: emissivity = absorptivity = 1 −  reflectivity. The measured reflectivity at 2.4 eV as a function of time is displayed in Fig. 1(b) for Au only and (c) for Al targets. The reflectivity beyond the probe time window is extrapolated until it reaches zero. The slight difference in the wavelengths in the two diagnostics (photon energy 2.4 eV in FDI vs 3.1 eV in SOP) has an insignificant effect based on broadband optical measurements in the literature^36, 37^. For Al, both wavelengths are far away from any transitions and the spectral response is fairly flat from 2.4 eV to 3.1 eV^36^, hence we used ${R}_{3.1eV}(t)={R}_{2.4eV}(t)$. For Au, both wavelengths are above an interband transition and show similar trend upon heating^37^, hence we adopted ${R}_{3.1eV}(t)={R}_{3.1eV}(t=0)\times {R}_{2.4eV}(t)/{R}_{2.4eV}(t=0)$. Furthermore, the finite emissivity correction is only important at early time up to ~15 ps. After that, the reflectivity is low so that the system behaves close to a blackbody. The error bar is assessed to be ±15% at 0–15 ps and ±10% at later time.

### Simulations

The competing processes of heat flow from thermal conduction and cooling from surface expansion necessitates employment of hydrodynamic simulations as our primary tool for assessing various thermal conductivity models. The simulations were performed in a 1D cartesian geometry with HYDRA^38^ – a multi-physics, multi-dimensional, arbitrary Lagrangian-Eulerian, radiation-hydrodynamics code. The on-shot proton energy spectrum from the Thomson parabola was used as the heating source since the stopping by the ultrathin multilayer target is negligible compared to the MeV proton energies. The 50 *μm* vacuum gap between the proton-generating foil and the main multilayer target resulted in a heating duration of ~8 *ps* due to the energy-dependent time-of-flight. The only fitting parameter in the simulations is a source multiplier to take into account finite divergence of the proton beam (see Methods). This parameter is determined by the Au-only data and kept the same for all other shots for consistency.

The sensitivity of our observables to many hydrodynamic processes is investigated by systematically varying the corresponding properties in HYDRA simulations. It is confirmed that properties of the *Si*_3_ *N*_4_ layer have little effect on the measurements at the Al side. We also found that varying the electron-ion equilibration rates in Au and Al by two orders of magnitude results in insignificant change in the measured thermal emission and phase shift. A contamination layer at the Al surface^39^ has negligible effect on the observables as well. Even the thermal conductivity of Au has very small effect on the data. This is understandable as Au thermal conductivity is much higher than Al, hence the heat flow rate is restricted mainly by the “slower conductor” Al. We have identified the properties that play a major role in the measurements are the equation of state (EOS) and Al thermal conductivity under our experimental conditions.

### Au release EOS

**Figure 2:**
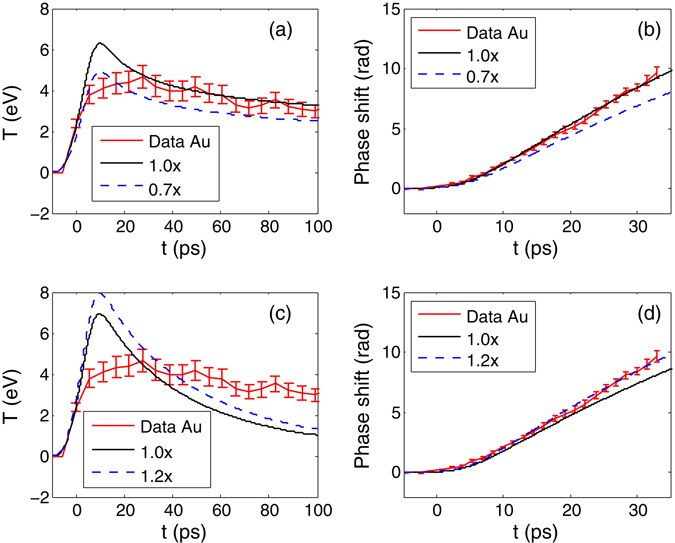
Comparison of data for Au-only targets and HYDRA simulation outputs to constrain the Au EOS. The time history of the temperature is from SOP and the phase shifts is from FDI measurements. (**a**) and (**b**) show results using LEOS L790, and (**c**) and (**d**) using Sesame table 2700 for Au EOS. The number in the legend is a multiplier in the proton heating source.

Figure 2 shows the measured temperature and phase shift vs time for Au-only targets along with simulations using LEOS^40, 41^ L790 and Sesame^42^ 2700 for Au EOS. The LEOS results match the temperature data quite well at 15–100 ps, and also agree with the phase data where the data is available (0–30 ps). The early time discrepancy in the temperature also exists in the case of Al, which will be discussed later. It is clear from Fig. 2(a) and (c) that the source multiplier affects the peak temperature, but not the temporal slope in the temperature. Therefore it is easy to determine the source multiplier from the SOP data only. The same multiplier also provides good agreement with the Au FDI data using L790 as shown in Fig. 2(b). On the other hand, the simulation with Sesame 2700 predicts a temperature evolution very different from the measured time history. In order to match the phase shift, the multiplier needs to be 20% higher than the L790 case. No matter how the source multiplier is changed, Sesame 2700 predictions do not agree with the Au SOP data. Therefore, we choose to use L790 as the Au EOS in the following simulations.

This difference in EOS response is understandable given the very different temperature dependence in the two EOS models. Although the two models have similar Hugoniots below 1 Mbar, they diverge notably above that pressure, and also have very different off-Hugoniot response. Assuming isochoric heating, the same energy input of 10 kJ/g would result in LEOS 790 with a pressure of 1.9 Mbar and temperature 3.5 eV while Sesame 2700 would have a pressure of 1.6 Mbar and a temperature of 3.85 eV.

### Al release EOS

**Figure 3:**
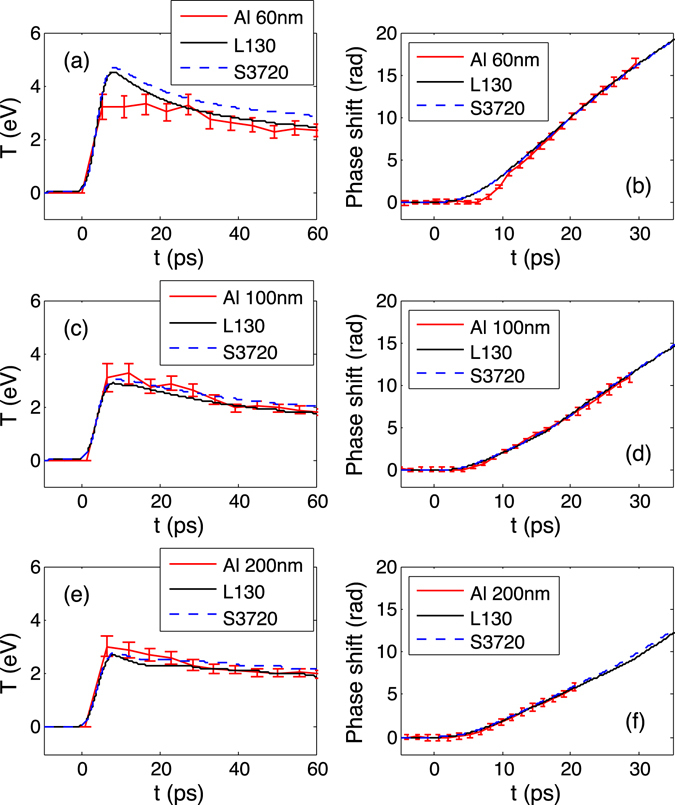
Comparison of 100 nm Au +60 nm (**a**) /100 nm (**c**) /200 nm (**e**) Al SOP data with HYDRA simulations using LEOS L130 and Sesame 3720 for Al EOS, and Purgatorio for the thermal conductivity. (**b**,**d**,**f**) are corresponding comparisons with the FDI data.

Similar to the Au EOS, a comparison of Al data and simulations using LEOS L130 and Sesame 3720 is shown in Fig. 3 for three Al thicknesses 60, 100, and 200 nm. For the two thicker cases, both EOS tables are consistent with the data within the error bars. In the case of 60 nm Al, Sesame 3720 predicts a temperature higher than the data, and the temperature from L130 is at the upper limit of the error bars. Therefore, L130 is preferred in the simulations. Early time discrepancy is observed in both SOP and FDI data for Al 60 nm. Because thermal conduction is a much longer process than the heating, thermal conductivity models can still be tested based on late time behaviors, which is confirmed by varying thermal conductivity in the HYDRA simulations.

### Al thermal conductivity

**Figure 4:**
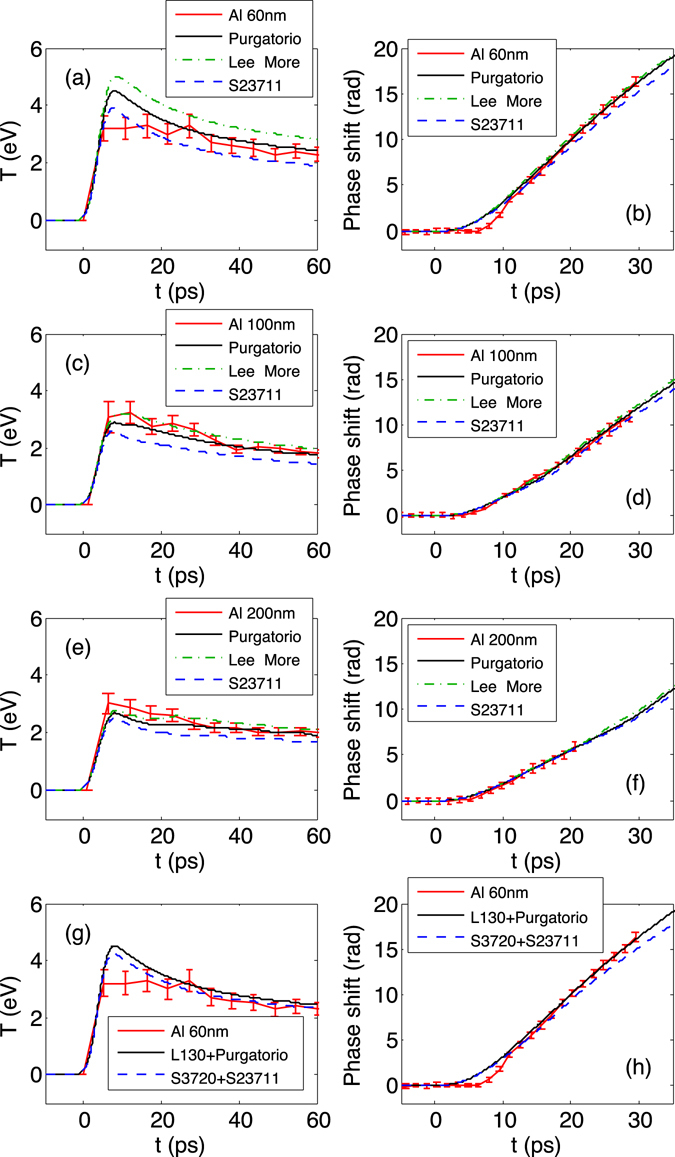
Comparison of 100 nm Au + 60 nm (**a**) /100 nm (**c**) /200 nm (**e**) Al SOP data with HYDRA simulations using L130 for Al EOS, Purgatorio, Lee-More and Sesame 23711 for Al thermal conductivity. (**b**,**d**,**e**) are corresponding comparisons with the FDI data. (**g**,**h**) are 100 nm Au + 60 nm Al data and simulations including Sesame 3720 for Al EOS and Sesame 23711 for Al thermal conductivity.

With the EOS constrained, we look at how commonly used thermal conductivity models for Al in this regime compare with the Al data. Three representative models that are available for HYDRA simulations, Sesame 23711, Lee-More^3^, and Purgatorio^8^, are compared in Fig. 4. The Lee-More model has a free parameter *γ* that is chosen to be 1.8 to provide Al thermal conductivity in agreement with those presented in the original Lee-More paper^3^. For the case of 60 nm Al shown in Fig. 4(a) and (b), the temperature predicted by the Lee-More is too high compared to the SOP data; The Sesame 23711 prediction is close to the lower limit of the SOP data, however the phase shift is outside the FDI data error bar; Purgatorio predictions are in reasonable agreement with both SOP and FDI data. For the 100 nm and 200 nm Al, both Lee-More and Purgatorio predictions are within the data error bars. The Sesame 23711 provides a temperature 20–30% lower than the other two models and also lower than the data, indicating a too low thermal conductivity by this model.

Since Sesame EOS 3720 overpredicts the temperature and Sesame thermal conductivity table 23711 underpredicts the temperature, we also performed simulations using these two Sesame tables for the EOS and thermal conductivity, respectively. This set of simulation results are displayed for the case of 60 nm Al in Fig. 4(g) and (h). Although the temperature agreement is reasonable, the simulated phase shift is too low compared to the data.

The multiple data sets, including Au only and three Al thicknesses, are all time-resolved and consist of about 80 effective data points, providing strong constraints on both Au/Al EOS and Al thermal conductivity in our case. We have tested all possible combinations of the two Al EOS tables (L130 and S3720) and five thermal conductivity models (Lee-More, Purgatorio, S23711, S23714 and S29373) that we have access to. Only L130 with Purgatorio or S23714 thermal conductivity can match all Al data after 15 ps.

### Density-temperature regions

**Figure 5:**
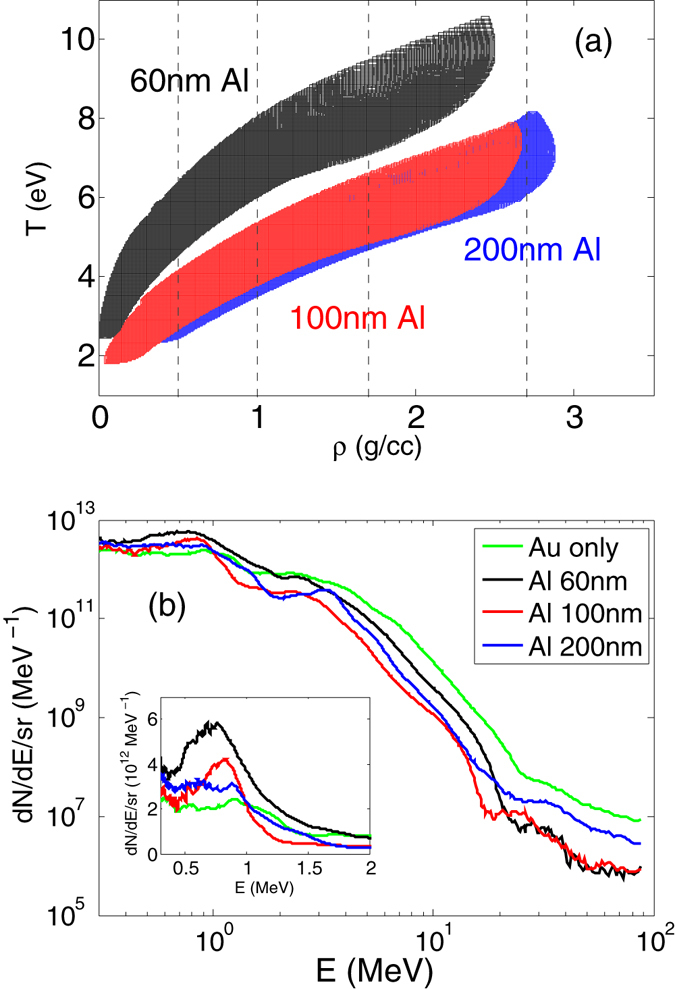
(**a**) Density-temperature range from simulations. (**b**) Proton energy spectra.

Because thermal conduction only occurs when there is a temperature gradient, this experiment does not provide single-state measurements of thermal conductivity. To find out the density-temperature range explored in this experiment, we examined the evolution path of density and temperature in Al from simulations that match all the Al data. The results are plotted in Fig. 5(a). Clearly the paths are very similar for the 100 nm and 200 nm Al, and the 60 nm Al shot evolves along a higher-temperature path. The corresponding proton energy spectra are shown in Fig. 5(b), including the Au-only shot. On a log-scale, the spectra look quite similar. On the linear scale as in the inset plot, the 60 nm Al shot has the highest proton flux in 0.5–1.5 MeV range which is near the highest stopping power of protons in Au and Al^30^ thus most effective in heating. This explains that the higher temperature in the 60 nm Al shot compared to 100 and 200 nm Al shots was due to single shot variation of the proton source as shown in Fig. 5(b).

To quantify the Al thermal conductivity and narrow down the corresponding density-temperature regions, we divided the density into three regions, 0.5–1.0 g/cc, 1.0–1.7 g/cc, and 1.7–2.7 g/cc, as marked by vertical dashed line in Fig. 5(a), and applied a local multiplier to the thermal conductivity in each specific density-temperature region in HYDRA simulations to investigate the sensitivity. The multiplier is varied to match the upper and lower limits in both SOP and FDI data. It is found that for 100 nm and 200 nm Al, the thermal conductivity can be constrained to 0.5 × −1.5× of Purgatorio values, while for 60 nm Al, the result is 0.3 × −1.0× of Purgatorio values. We also tested the sensitivity at densities below 0.5 g/cc and found that our observables are not sensitive to thermal conduction in this low-density region. This is understandable as this low-density region has an electron density comparable or less than the critical density *n*_*c*_ for 400 nm. The thermal emission at 400 nm is mainly generated near *n*_*c*_ because above *n*_*c*_ the optical emission cannot escape, and below *n*_*c*_ the temperature is lower hence the emission is much less. As the heat flows from the hotter Au toward the Al rear surface, it is probed by the 400 nm emission near *n*_*c*_. The thermal conduction downstream has insignificant effects on the temperature near *n*_*c*_. As a result, our observables are not sensitive to the thermal conductivity below 0.5 g/cc.

**Figure 6:**
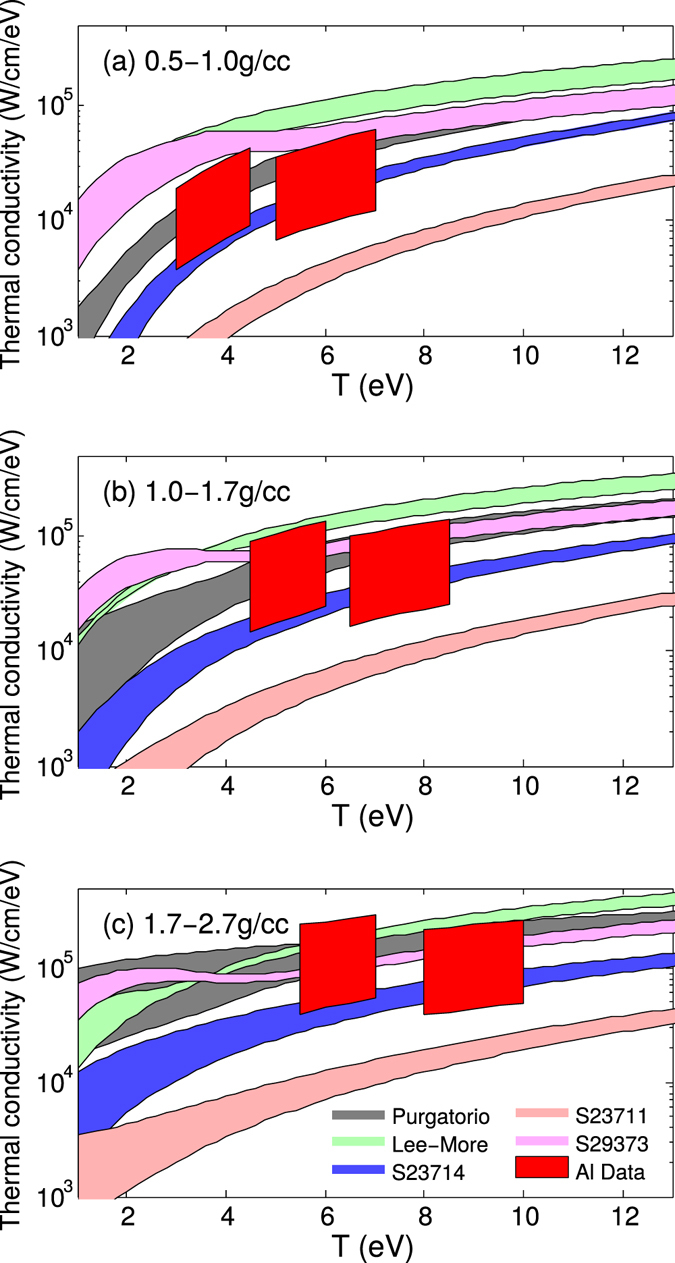
Comparison of five thermal conductivity models with data in three density ranges.

The Al thermal conductivity as a function of temperature in the three density regions is shown in Fig. 6. It can be seen that in these density and temperature ranges, thermal conductivities by the Lee-More model is higher than the Purgatorio model, and the Sesame 23711 tables predicts a much lower thermal conductivity, consistent with Fig. 4(a–f). Two more models, Sesame 23714 and 29373 are also plotted in Fig. 6. The thermal conductivities constrained by our data are displayed as red areas. At 0.5–1.0 g/cc only Purgatorio and S23714 agree with the data, and at 1.0–2.7 g/cc, three models, Purgatorio, S23714 and S29373, agree with the data. The error bars in the data are about a factor of 4–6, which is comparable to the error bars in the recent electrical conductivity measurements^26^.

Both Sesame 23711 and 23714 are based on the Rinker model^6^, using the Born approximation in 23711^6^ and the extended Ziman formula^43^ in 23714^7^. From Fig. 6 it is clear that S23714 is more accurate and much closer to the data than S23711. Sesame 29373 employed Lee-More-Desjarlais modification to the Lee-More model based on quantum molecular dynamics simulations^4, 5^, which is an approach totally different from Purgatorio and Rinker but provided comparable thermal conductivity to Purgatorio in the density-temperature region explored here. At 4–10 eV, it is generally expected that the thermal conductivity of Al increases with temperature. The difference between the two data points is much smaller than the error bar, hence insufficient to test the temperature trend. To further discriminate between Purgatorio and S23714, and to study the temperature dependence, experiments with smaller error bars and narrower density-temperature regions are required. Nonetheless, our measurements provide the first benchmark for models on thermal conductivity of warm dense aluminum.

## Discussion

As described above, discrepancy exists at early time. There could be a few reasons for this discrepancy. First of all, it is known that during the heating, the system can be highly non-equilibrium. Models under non-equilibrium conditions have not been fully developed yet and discrepancy has been observed in other experiments^44^. In addition, most hydrodynamic properties are defined under equilibrium conditions. Thermal conductivity is not well-defined if the temperature is not well defined in cases such as non-Maxwellian velocity distributions^45^. Another uncertainty at early time is the emissivity correction since the reflectivity was measured at 2.4 eV and the thermal emission were measured at 3.1 eV although we expect that this has an insignificant effect. Furthermore, phase transitions such as melting can occur during early times, which is not well modeled in hydrodynamic codes. All these topics are active research areas but are beyond the scope of this paper.

To ensure the thermal emission collected by SOP is from Al not from Au, we have included radiation transport in the simulations as described in Methods. It is confirmed that although the target layers have substantially expanded in the time window of the measurements, the Al opacity is high enough to completely absorb the Au emission even for the thinnest 60 nm Al so that the detected emission is from Al thus providing information on the heat transport in Al.

There is a material interface in our targets, diffusion can lead to material mix near the interface which is not included in HYDRA simulations but could complicate the data interpretation. We have estimated the diffusion length of Au into Al under our experimental conditions using plasma-based kinetic theory^46^, which provided diffusivities comparable to recent molecular dynamics calculations^47^, hence is good enough for a reasonable estimate. The inter-diffusivity is given by ${D}_{12}=\frac{3{k}_{B}{T}_{i}}{16{n}_{i}{m}_{redu}{{\rm{\Omega }}}_{12}}$ ^48^, where *k*_*B*_ is Boltzman constant, *T*_*i*_ is ion temperature, *n*_*i*_ is ion density, ${m}_{redu}=\frac{{m}_{1}{m}_{2}}{{m}_{1}+{m}_{2}}$ is the reduced mass of particles with mass *m*_1_ and *m*_2_, and Ω_12_ is the first collision integral which can be derived analytically for a pure Coulomb interaction^46^. Using the density, temperature and ionization stages near the Au/Al interface in the HYDRA simulations, the inter-diffusivity of Au into Al is *D*_12_  ~ 4 × 10^−7^ *m* ^2^/*s* at 10 ps and 8 × 10^−7^ *m* ^2^/*s* at 60 ps. The higher diffusivity at later time is mainly resulted from lower density due to target expansion. Taking an average value 6 × 10^−7^ *m* ^2^/*s*, the spatial scale of the diffusion evolves over time as $\sqrt{4{D}_{12}t} \sim 12\,nm$ at 60 ps. Our Al target initial thickness is 60, 100 and 200 nm, already much larger than the estimated diffusion length. Therefore, we do not expect diffusion to play a role in our measurements. Another possible process at the interface is contact resistance, which is induced by granular nature of interfaces. Our targets were fabricated in vacuum and have optical quality surfaces that is required for the FDI measurements. Therefore, we do not expect the contact between the two materials to be an issue in our experiments.

## Methods

The optical system for SOP was calibrated *in situ* with a 404 nm CW laser and the streak camera was calibrated using 100 fs Europa laser pulses at 400 nm at JLF. The temporal resolution of the streak camera was measured to be 6 ps at the same sweep speed for data acquisition. The wavelength-dependent response of the SOP system within the 70 nm bandwidth was provided by the manufacturers. Both SOP and FDI are spatially resolved in one dimension. The measured heated region is ~300 *μm* in diameter. The central 70 *μm* region where the profile is flat is averaged to provide the time history of temperature and phase shift. The large ratio of transverse (70 *μm*) vs the longitudinal (up to 0.4 *μm*) ensures a 1D geometry.

In the HYDRA simulations, the proton source was modeled using the inline heavy ion beam deposition model, where protons travel and deposit energy along rays similar to those used by the 3D laser package^49^. We used the built-in stopping power model, based on the work of Betz^50^, which employs a modified Bethe-Bloch formulation for ion energy deposition^51, 52^. It is known that TNSA proton beams have a divergence of 40–60°. The central 70 *μm* region relative to the 300 *μm* heated area corresponds to a divergence angle of 10–15°. The source multiplier in the simulations is 1.0 if assuming a divergence of 14°.

Radiation transport was included in the simulations using multi-group, implicit Monte Carlo (IMC) photonics^53^ with opacity tables generated by Livermore’s online opacity server. To directly compare with the SOP data, emission near 400 nm from the target was calculated and spectrally resolved using the emissivity and opacity tables in the HYDRA simulations. The effective temperature is obtained at each time step so that a Planckian spectrum at this temperature produces the same counts on the SOP detector as the simulated emission spectra after transmission through the whole calibrated optical system. The simulated temperature depends on the Al emissivity/opacity model in the underdense plasma region where the 400 nm thermal emission transports through. We have also compared this effective temperature with the temperature at the critical density *T*_*nc*_. In case of Au only, the expansion is small and these two temperature agree well in the time window of interest. For Al, the effective temperature is ~10–20% lower than *T*_*nc*_ and in better agreement with data.

### Data Availability

The datasets generated during and/or analyzed during the current study are available from the corresponding author on reasonable request.
